# Causes of Morbidity in Wild Raptor Populations Admitted at a Wildlife Rehabilitation Centre in Spain from 1995-2007: A Long Term Retrospective Study

**DOI:** 10.1371/journal.pone.0024603

**Published:** 2011-09-23

**Authors:** Rafael A. Molina-López, Jordi Casal, Laila Darwich

**Affiliations:** 1 Centre de Fauna Salvatge de Torreferrussa, Catalan Wildlife-Service, Forestal Catalana, Spain; 2 Departament de Sanitat i Anatomia Animals, Faculty of Veterinary, Universitat Autònoma de Barcelona, Barcelona, Spain; 3 Centre de Recerca en Sanitat Animal, UAB-IRTA, Campus Universitat Autònoma de Barcelona, Barcelona, Spain; University of Georgia, United States of America

## Abstract

**Background:**

Morbidity studies complement the understanding of hazards to raptors by identifying natural or anthropogenic factors. Descriptive epidemiological studies of wildlife have become an important source of information about hazards to wildlife populations. On the other hand, data referenced to the overall wild population could provide a more accurate assessment of the potential impact of the morbidity/mortality causes in populations of wild birds.

**Methodology/Principal Findings:**

The present study described the morbidity causes of hospitalized wild raptors and their incidence in the wild populations, through a long term retrospective study conducted at a wildlife rehabilitation centre of Catalonia (1995–2007). Importantly, Seasonal Cumulative Incidences (SCI) were calculated considering estimations of the wild population in the region and trend analyses were applied among the different years. A total of 7021 birds were analysed: 7 species of Strigiformes (n = 3521) and 23 of Falconiformes (n = 3500). The main causes of morbidity were trauma (49.5%), mostly in the Falconiformes, and orphaned/young birds (32.2%) mainly in the Strigiformes. During wintering periods, the largest morbidity incidence was observed in *Accipiter gentillis* due to gunshot wounds and in *Tyto alba* due to vehicle trauma. Within the breeding season, *Falco tinnunculus* (orphaned/young category) and *Bubo bubo* (electrocution and metabolic disorders) represented the most affected species. Cases due to orphaned/young, infectious/parasitic diseases, electrocution and unknown trauma tended to increase among years. By contrast, cases by undetermined cause, vehicle trauma and captivity decreased throughout the study period. Interestingly, gunshot injuries remained constant during the study period.

**Conclusions/Significance:**

Frequencies of morbidity causes calculated as the proportion of each cause referred to the total number of admitted cases, allowed a qualitative assessment of hazards for the studied populations. However, cumulative incidences based on estimated wild raptor population provided a more accurate approach to the potential ecological impact of the morbidity causes in the wild populations.

## Introduction

Birds of prey are valuable sentinels of environmental changes because of their position at the top of the ecological food chain and because they are widespread across large geographical areas. In addition, they are particularly sensitive to ecological changes at a range of spatial scales [1], [2] and, as such, some species of free-living birds of prey and owls have decreased in numbers and become threatened or even endangered around the world. In fact, in Europe, 36 species (64%) of the total 56 different raptor species have an unfavourable conservation status [3].

Morbidity studies complement the understanding of hazards to raptors by identifying natural or anthropogenic factors. Therefore, the analysis of morbidity and mortality reports of free-living raptors presented to rehabilitation centres has provided insight into the primary and secondary causes, as well as in the evaluation of the health status of wild populations [4], [5]. However, there are few studies on morbidity in wild raptors of Spain, and these have focused on a limited number of species or specific causes [6]–[10]. In addition, global epidemiological studies of wild raptor diseases are also scarce, especially long term studies [11], [12]. Finally, while the information reported by such studies is critical for the rehabilitation centres management, this information has been mainly based on the proportion of cases in the total number of admissions at the centre. Only rarely have the data been referenced to the overall wild population, that could provide a more accurate assessment of the potential impact of the morbidity/mortality causes in populations of wild birds.

The purpose of this study was to analyze the causes of morbidity in a large population of raptors admitted at one rehabilitation centre in Spain from 1995 to 2007 using specific epidemiological data (species, gender, age, season, and year) as well as the Seasonal Cumulative Incidences (SCI) considering estimations of the wild population in the region for the different raptor species.

## Results

### Descriptive analyses

A total of 7553 admission reports were reviewed. Of those, 532 cases were excluded for not fulfilling the inclusion criteria. Thus, the final study population was 7021 individuals homogenously distributed in two orders: Order Strigiformes with 3521 animals corresponding to seven species of owls and Order Falconiformes with 3500 animals of 23 different diurnal raptor species. The majority of animals (89.5%, n = 6282) were alive when admitted. Within the species represented in the study, there were some important species catalogued as “in danger of extinction” (*Gypaetus barbatus*) and “vulnerable” (*Circus pygargus, Achila fasciata, Milvus milvus, Neophron percnopterus and Pandion haliaetus*) by the Spanish Catalogue of Menaced Species [13].

Most of the animals, 58.7% (n = 4119), were classified as undetermined gender, 22.5% (n = 1579) of raptors were sexed as female (F) and 18.8% (n = 1323) as males (M). Within the undetermined gender group, the majority of birds belonged to the Strigiformes order, representing 67% (2746/4119) of birds; the remaining 33% (1373/4119) of undetermined sex belonged to the Falconiformes order. Only three species -*Achila fasciata, Accipiter nisus* and *Otus scops-* showed significant differences between genders with ratios of 6F/15M (χ ^2^ = 105, *P* = 0.0001), 329F/96M (χ^2^ = 4.69, *P* = 0.03) and 91F/61M (χ ^2^ = 12.58, *P* = 0.0004), respectively.

**Figure 1:**
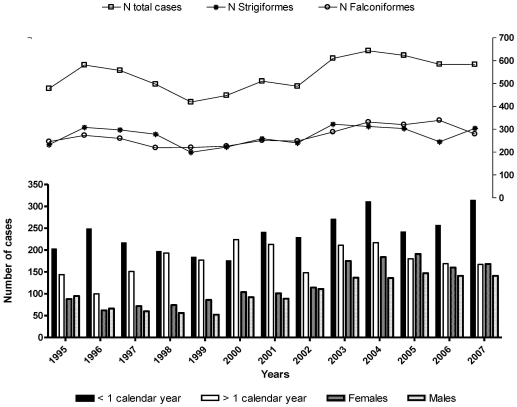
Admissions of birds of prey stratifying by raptor order, age and sex, yearly distributed along the period 1995-2007.

The age distribution showed that 44% (3091/7021) of birds were within the first year calendar, 32.7% (2294/7021) > 1 year calendar and 23.4% (1636/7021) were of unknown age (Table 1). The dynamic of cases throughout the study period showed a homogenous entry of cases per year (ranging from 478 to 643 cases), with similar number of cases of raptors by order, gender and age among the different years (Fig. 1).

### Distribution of primary causes of morbidity

**Table 1:** Frequency of admission in the rehabilitation centre and demographic data of raptors included in the study during the period 1995-2007.

Species descriptive: Common name (*scientific name*)	Cases	Sex		Age (one year calendar)		
Order Strigiformes	Number	F/M*	Unknown	<1 year	>1 year	Unknown
**Family Tytonidae**						
Common barn owl (*Tyto alba*)	500	81/74	345	157	174	169
**Family Strigidae**						
Eurasian scops owl (*Otus scops*)	878	61/91	726	655	129	94
Eurasian eagle-owl (*Bubo bubo*)	198	54/62	82	28	110	60
Tawny owl (*Strix aluco*)	731	56/63	612	475	168	88
Little owl (*Athene noctua*)	1120	98/107	915	729	220	171
Northern long-eared owl (*Asio otus*)	82	18/7	57	19	25	38
Short-eared owl (*Asio flammeus*)	12	2/1	9	0	6	6
**Order Falconiformes**						
**Family Pandionidae**						
Osprey (*Pandion haliaetus*)	6	1/2	3	1	3	2
**Family Accipitridae**						
Western Honey-buzzard (*Pernis apivorus*)	61	12/8	41	19	22	20
Red kite (*Milvus milvus*)	7	1/6	17	1	4	2
Black kite (*Milvus migrans*)	24	1/1	5	6	7	11
Bearded vulture (*Gypaetus barbatus*)	2	0/2	0	0	2	0
Egyptian vulture (*Neophron percnopterus*)	2	0/0	2	1	1	0
Eurasian griffon (*Gyps fulvus*)	49	2/4	43	16	17	16
Short-toed Snake-eagle (*Circaetus gallicus*)	52	10/10	32	3	34	15
Western Marsh-harrier (*Circus aeruginosus*)	38	20/10	8	3	22	13
Hen harrier (*Circus cyaneus*)	14	6/4	4	0	11	3
Montagu's harrier (*Circus pygargus*)	13	3/8	2	8	5	0
Eurasian Sparrowhawk (*Accipiter nisus*)	466	329/96	41	103	227	136
Northern Goshawk (*Accipiter gentillis*)	231	108/84	39	93	106	32
Eurasian buzzard (*Buteo buteo*)	934	245/210	479	71	413	450
Golden eagle (*Aquila chrysaetos*)	7	3/3	1	1	5	1
Bonelli's eagle (*Aquila fasciata*)	31	6/15	10	6	16	9
Booted eagle (*Aquila pennata*)	30	5/10	15	4	17	9
**Family Falconidae**						
Lesser kestrel (*Falco naumanni*)	88	28/27	33	54	26	8
Common kestrel (*Falco tinnunculus*)	1295	382/361	552	591	451	253
Red-footed falco (*Falco vespertinus*)	2	1/1	0	0	2	0
Merlin (*Falco columbarius*)	7	3/3	1	0	4	3
Eurasian hobby (*Falco subbuteo*)	35	5/10	20	3	21	11
Peregrine falcon (*Falco peregrinus*)	106	38/43	25	44	46	16
**Total**	**7021**	**1579/1323**	**4119**	**3091**	**2294**	**1636**

*F/M, female/male ratio.

The two most frequent causes of admission were trauma (49.5%; 95% CI: 48.3–50.7) and orphaned young birds (32.2%; 95% CI: 31.1–33.3). The other primary causes had frequencies below 10% (Table 2). Trauma was more frequently observed in Falconiformes. This order showed the highest risk of gunshot or electrocution. Risks of falling into traps, power lines or being predated were similar between both raptor orders and traumas with motor vehicles and fences were considerably higher in nocturnal raptors likely due to their habit of hunting along roads and their feature to be easily dazzled (Table 2). It is interesting to note that owls and *Falco tinnunculus* (χ^2^ = 21.39, *P*<0.0001) represented the largest group of animals found inside buildings (Table 3), while most of *Accipiter gentillis* birds were captured inside chicken farms (χ^2^ = 153.70, *P*<0.0001). Trichomoniasis was the most frequent cause of infectious/parasitic disease with positive cases in the following species: *Falco tinnunculus* (19 cases), *Strix aluco* (7), *Tyto alba* (5), *Accipiter gentillis* (5), *Falco peregrinus* (4), and *Accipiter nisus*, *Circus pygargus*, *Bubo bubo* and *Achila fasciata* with 1 case each, respectively (Table 2). Fatal intoxication was diagnosed in: *Gyps fulvus* for lead toxicity (1), *Tyto alba* for Bromodiolone (2), *Buteo buteo* (2) and *Circaeuts gallicus* (1) for carbofuran, and *Falco naumanni* for cipermetrine (1).

**Table 2:** Frequency of primary causes of admission and statistical comparison between Strigiformes and Falconiformes orders.

	Overall Prevalence		Strigiformes (N = 3521)		Falconiformes (N = 3500)		Odds Ratio (OR)	
Primary Causes	Total number	Percentage (95% CI)	Total number (%)	Percentage	Total number (%)	Percentage	OR (CI 95%)	
**Trauma:**	3476	49.5 (48.3–50.7)	1182 (33.6)	100	2294(65.5)	100	0.3 (0.2–0.3)	<0.0001
Unknown	1817	25.9 (24.8–26.9)	694 (19.7)	58.7	1123 (32.1)	49	1.4 (1.2–1.7)	<0.0001
Gunshot	689	9.8 (9.1–10.5)	48 (1.4)	4.1	641 (18.3)	27.9	0.1 (0.08–0.1)	<0.0001
Vehicles	571	8.1 (7.5–8.7)	322 (9.1)	27.2	249 (7.1)	10.9	3.0 (2.5–3.6)	<0.0001
Electrocution	281	4.0 (3.5–4.5)	60 (1.7)	5.1	221 (6.3)	6.3	0.5 (0.3–0.6)	<0.0001
Buildings	58	0.8 (0.6–1)	28 (0.8)	2.4	30 (0.9)	1.3	1.8 (1.1–3.1)	<0.010
Traps	19	0.3 (0.2–0.4)	5 (0.1)	0.4	14 (0.4)	0.6	0.7 (0.2–1.9)	ns
Fences	24	0.3 (0.2–0.5)	21 (0.6)	1.8	3 (0.1)	0.2	13.8 (4.1–46.4)	<0.0001
Power lines	11	0.2 (0.1–0.3)	2 (0.1)	0.2	9 (0.3)	0.4	0.4 (0.1–1.9)	ns
Predation	6	0.1 (0.05–0.2)	2 (0.1)	0.2	4 (0.1)	0.2	0.9 (0.1–5.3)	ns
**Orphaned young**	2260	32.2 (31.1–33.29)	1768 (50.2)	100	492 (14.1)	100	6.1 (5.4–6.9)	<0.0001
**Fortuity:**	398	5.7 (5.1–6.2)	249 (7.1)	100	149 (4.3)	100	1.7 (1.3–2.1)	<0.0001
Buildings	289	4.1 (3.6–4.6)	191	76.7	98	65.8	1.7 (1.1–2.6)	0.0179
Others^a^	65	0.9 (0.7–0.2)	37	14.9	28	18.8	0.7 (0.4–1.3)	ns
Water ponds	44	0.6 (0.4–0.8)	21	8.4	23	15.4	0.5 (0.2–0.9)	0.0311
**Undetermined**	379	5.4 (4.8–5.9)	161 (4.6)	100	218 (6.2)	100	0.7 (0.5–0.8)	<0.005
**Metabolic/nutritional:**	235	3.3 (2.9–3.8)	76 (2.2)	100	159 (4.5)	100	0.4 (0.3–0.6)	<0.0001
Emaciation	151	2.1 (1.8–2.5)	52	68.4	99	62.3	1.3 (0.7–2.3)	ns
Others^b^	48	0.6 (0.5–0.9)	16	21.1	32	20.1	1.4 (0.7–2.6)	ns
MBD	36	0.5 (0.3–0.7)	8	10.5	28	17.6	0.5 (0.2–1.2)	ns
**Captivity**	158	2.3 (1.9–2.6)	47 (1.3)	100	111 (3.2)	100	0.2 (0.1–0.2)	<0.0001
**Infectious/parasitic:**	108	1.5 (1.2–1.8)	34(1)	100	74(2.1)	100	0.4 (0.2–0.6)	<0.0001
Others^c^	55	0.7 (0,5–1)	21	61.8	34	45.9	1.9 (0.8–4.3)	ns
Trichomoniosis	44	0.6 (0,4–0.8)	13	38.2	31	41.9	0.8 (0.3–1.9)	ns
**Toxicoses**	7	0.1 (na)	4 (0.1)	100	3 (0.1)	100	1.3 (0.3–5.9)	ns

CI: confidence interval. ns: no statistical significance (p>0.05). na: not applicable. MBD, metabolic bone diseases. Others:

a, manure heaps, bad weather;

b, rest of diagnoses grouped by organic systems such as musculoskeletal, digestive, nervous, integument, and ocular diseases;

c, mycobacteriosis, helminthiasis, mites, abscess.

**Table 3:** Number of cases and frequency distribution by primary causes of admission and species.

	TRAUMA: Number of cases (%)									OTHERS: Number of cases (%)							
Species	Unknown	Gunshot	Vehicles	Electrocuted	Building	Fences	Traps	PowerLines	Predation	Orphaned young	Fortuity	Undetermined	Metabolicdiseases	Captivity	Infectiousdiseases	Toxicity	Total
*Accipiter gentillis*	65 (28)	77 (33)	4 (2)	12 (5)	1 (0)	0	1 (0)	0	1 (0)	11 (5)	25 (11)	16 (7)	5 (2)	8 (4)	5 (2)	0	**231**
*Accipiter nisus*	224 (48)	122 (26)	26 (6)	1 (0)	20 (4)	2 (0)	1 (0)	1 (0)	0	12 (3)	11 (2)	27 (6)	7 (1)	5 (1)	7 (2)	0	**466**
*Aquila chrysaetos*	3 (43)	0	0	1 (14)	0	0	0	0	0	0	0	3 (43)	0	0	0	0	**7**
*Asio flameus*	6 (50)	6 (50)	0	0	0	0	0	0	0	0	0	0	0	0	0	0	**12**
*Asio otus*	37 (45)	7 (8)	9 (11)	1 (1)	0	2 (2)	0	0	0	13 (16)	5 (6)	4 (5)	4 (5)	0	0	0	**82**
*Athene noctua*	255 (23)	16 (1)	105 (9)	4 (0)	6 (1)	5 (0)	3 (0)	0	0	585 (52)	59 (5)	43 (4)	16 (1)	18 (2)	5 (1)	0	**1120**
*Bubo bubo*	49 (25)	8 (4)	16 (8)	48 (24)	0	10 (5)	0	2 (1)	0	13 (7)	20 (10)	12 (6)	14 (7)	3 (2)	3 (2)	0	**198**
*Buteo buteo*	279 (30)	272 (29)	138 (15)	79 (8)	0	0	1 (0)	3 (0)	1 (0)	16 (2)	26 (3)	62 (7)	32 (3)	13 (1)	11 (1)	1 (0)	**934**
*Circaetus gallicus*	18 (35)	2 (4)	3 (6)	15 (29)	0	1 (2)	0	2 (4)	0	1 (2)	1 (2)	5 (10)	2 (4)	0	1 (2)	1 (2)	**52**
*Circus aeruginosus*	17 (45)	6 (16)	0	0	0	0	0	0	0	0	4 (10)	6 (16)	4 (11)	0	1 (3)	0	**38**
*Circus cyaneus*	6 (43)	6 (43)	0	0	0	0	0	0	0	1 (7)	0	1 (7)	0	0	0	0	**14**
*Circus pygargus*	7 (54)	1 (8)	0	0	0	0	0	0	0	0	0	1 (8)	1 (8)	0	3 (23)	0	**13**
*Falco columbarius*	4 (57)	2 (29)	0	0	0	0	0	0	0	0	1 (14)	0	0	0	0	0	**7**
*Falco naumanni*	21 (24)	0	4 (4)	0	0	0	0	0	2 (2)	22 (25)	2 (2)	14 (16)	9 (10)	12 (14)	1 (1)	1 (1)	**88**
*Falco peregrinus*	30 (28)	28 (26)	6 (6)	9 (8)	0	0	0	1 (1)	0	7 (7)	2 (2)	8 (7)	5 (5)	4 (4)	6 (6)	0	**106**
*Falco subbuteo*	15 (43)	6 (17)	2 (6)	1 (3)	0	0	0	0	0	0	2 (6)	2 (6)	2 (6)	2 (6)	3 (9)	0	**35**
*Falco tinnunculus*	373 (29)	89 (7)	54 (4)	87 (7)	6 (1)	0	11 (1)	0	0	415 (32)	63 (5)	54 (4)	45 (3)	66 (5)	32 (3)	0	**1295**
*Falco vespertinus*	0	0	0	0	1 (50)	0	0	0	0	0	0	1 (50)	0	0	0	0	**2**
*Gypaetus barbatus*	2 (100)	0	0	0	0	0	0	0	0	0	0	0	0	0	0	0	**2**
*Gyps fulvus*	6 (12)	3 (6)	2 (4)	0	0	0	0	0	0	2 (4)	0	2 (4)	33 (67)	0	1 (2)	0	**49**
*Hieraetus fasciatus*	7 (23)	4 (13)	0	5 (16)	0	0	0	1 (3)	0	0	2 (7)	8 (26)	2 (6)	0	2 (7)	0	**31**
*Hieraetus pennatus*	8 (27)	12 (40)	1 (3)	5 (17)	0	0	0	0	0	2 (7)	0	0	1 (3)	1 (3)	0	0	**30**
*Milvus migrans*	9 (37)	0	5 (21)	1 (4)	0	0	0	1 (4)	0	1 (4)	4 (17)	1 (4)	1 (4)	0	1 (4)	0	**24**
*Milvus milvus*	1 (14)	1 (14)	0	3 (43)	0	0	0	0	0	1 (14)	0	1 (14)	0	0	0	0	**7**
*Neophron percnopterus*	1 (50)	0	0	0	0	0	0	0	0	0	0	1 (50)	0	0	0	0	**2**
*Otus scops*	127 (14)	0	37 (4)	0	8 (1)	1 (0)	0	0	1 (0)	586 (67)	55 (6)	28 (3)	11 (1)	17 (2)	7 (1)	0	**878**
*Pandion haliaetus*	0	1 (17)	0	1 (17)	0	0	0	0	0	0	1 (17)	2 (33)	1 (17)	0	0	0	**6**
*Pernis apivorus*	27 (44)	9 (15)	4 (7)	1 (2)	2 (3)	0	0	0	0	1 (2)	5 (8)	3 (5)	9 (15)	0	0	0	**61**
*Strix aluco*	88 (12)	2 (0)	76 (10)	2 (0)	3 (0)	2 (0)	0	0	1 (0)	441 (60)	61 (8)	30 (4)	12 (2)	4 (1)	9 (1)	0	**731**
*Tyto alba*	132 (26)	9 (2)	79 (16)	5 (1)	11 (2)	1 (0)	2 (1)	0	0	130 (26)	49 (10)	44 (9)	19 (4)	5 (1)	10 (2)	4 (1)	**500**
**Total**	**1817**	**689**	**571**	**281**	**58**	**24**	**19**	**11**	**6**	**2260**	**398**	**379**	**235**	**158**	**108**	**7**	**7021**

No differences between genders related to any of the analyzed causes were observed (χ^2^ = 17.73, *P*>0.05). However, the first year calendar group had a higher risk of metabolic and nutritional diseases (OR = 3.7; 95%CI: 2.7–5.1), and infectious diseases (OR = 3.1; 95%CI: 1.95–4.85) compared to older birds. Conversely, the >1 year calendar group had a slightly higher risk of trauma with motor vehicles (OR = 1.36; 95%CI: 1.01–1.76) compared to the other age groups.

### Seasonality of specific causes of morbidity

A significantly higher number of cases were detected during the breeding period (χ^2^ = 1226.97, *P*<0.001), mainly due to orphaned young birds (Table 4). Metabolic or nutritional disease was significantly lower during the wintering season. Gunshot was concentrated during the autumn-winter hunting season (87.2%). Only 3.2% (22/689) of gunshot cases were recorded during the small game period at the end of August. The remaining 9.6% of cases (66/689) were detected out of hunting season. No statistically significant differences were observed among proportions of infectious/parasitic (χ^2^ = 1.76, *P*>0.05), fortuity (χ^2^ = 2.46, *P*>0.05) and electrocution (χ^2^ = 5.88, *P*>0.05) casualties.

**Table 4:** Intra-year distribution of primary causes of admission at the wildlife center according to seasonal periods (cases registered from 1995 to 2007).

Cause category	Breeding		Post-nuptial migration		Wintering		Total
	n	%	n	%	n	%	n
Orphaned young	1994	53.7	224	14.9	42	2.3	2260
Unknown trauma	672	18.1	503	33.5	642	35.5	1817
Gunshot	41	1.1	171	11.4	477	26.4	689
Motor vehicles	215	5.8	140	9.3	216	11.9	571
Fortuity	212	5.7	95	6.3	91	5.0	398
Undetermined	168	4.5	95	6.3	116	6.4	379
Electrocution	132	3.6	60	4.0	89	4.9	281
Metabolic/nutritional	102	2.7	103	6.9	30	1.7	235
Illegal	64	1.7	43	2.9	51	2.8	158
Infectious	60	1.6	26	1.7	22	1.2	108
Others (<100 cases)							
Trauma with building	23	0.6	20	1.3	15	0.8	58
Fences	8	0.2	6	0.4	10	0.6	24
Trap	9	0.2	6	0.4	4	0.2	19
Power lines	3	0.1	5	0.3	3	0.2	11
Intoxication	5	0.1	2	0.1	0	0.0	7
Predation	4	0.1	1	0.1	1	0.1	6
**Total**	**3712**	**100**	**1500**	**100**	**1809**	**100**	**7021**

Seasonal cumulative incidence (SCI) of the overall causes of admission regarding the main raptor species (those with at least 100 cases) are summarized in Table 5. The highest number of incidences during the wintering period was observed in *Accipiter gentillis* mainly due to gunshot and *Tyto alba* due to vehicle trauma. Species such as *Falco tinnunculus* (mainly due to orphaned young) and *Bubo bubo* (due to electrocution and metabolic disorders) represented the highest affected populations during the breeding season (Table 5).

**Table 5:** Seasonal incidence rate values of the different raptors species admitted during the 12 years of the study.

	Number of total cases^a^		Estimated^b^ population number		Overall causes^c^		Orphaned young^c^	Unknown trauma^c^		Gunshot^c^	Motor vehicles^c^		Fortuity^c^		Undetermined^c^		Electrocution^c^		Metabolic/nutritional^c^		Infectious/Parasitic^c^	
Raptor species*	W	B	W	B^d^	W	B	B	W	B	W	W	B	W	B	W	B	W	B	W	B	W	B
*Accipiter gentillis*	99	54	1438	750	5.74	0.73	0.15	1.39	0.20	2.78	0.12	0.01	0.64	0.08	0.46	0.05	0.17	0.08	0.00	0.03	0.06	0.01
*Accipiter nisus*	254	66	22954	1500	0.92	0.28	0.04	0.44	0.14	0.30	0.04	0.03	0.02	0.00	0.06	0.03	0.00	0.00	0.01	0.00	0.01	0.00
*Athene noctua*	112	844	5449	11669	1.71	0.67	0.65	0.81	0.11	0.18	0.24	0.04	0.11	0.03	0.06	0.03	0.02	0.00	0.03	0.01	0.02	0.00
*Bubo bubo*	56	95	1055	631	4.43	1.79	0.43	1.34	0.30	0.40	0.40	0.13	0.32	0.19	0.16	0.11	1.50	0.45	0.00	0.23	0.08	0.02
*Buteo buteo*	652	180	25710	1404	2.11	1.53	0.24	0.62	0.48	0.73	0.36	0.17	0.05	0.08	0.13	0.17	0.13	0.18	0.05	0.08	0.01	0.03
*Falco peregrinus*	37	36	1028	249	3.00	1.38	0.29	1.05	0.34	1.13	0.16	0.08	0.00	0.04	0.24	0.08	0.32	0.15	0.00	0.08	0.08	0.19
*Falco tinnunculus*	239	787	32003	3794	0.62	1.88	1.35	0.27	0.41	0.13	0.04	0.06	0.03	0.09	0.03	0.06	0.03	0.13	0.01	0.08	0.01	0.05
*Otus scops*	18	651	166	6515	9.06	0.93	0.98	2.01	0.12	0.00	0.50	0.03	0.50	0.06	0.00	0.03	0.00	0.00	0.00	0.01	0.00	0.01
*Strix aluco*	98	560	5981	13618	1.37	0.38	0.42	0.40	0.02	0.03	0.22	0.03	0.29	0.02	0.14	0.01	0.00	0.00	0.01	0.00	0.00	0.01
*Tyto alba*	124	240	1240	2765	8.34	0.76	0.52	3.16	0.13	0.34	2.22	0.08	0.74	0.07	0.94	0.04	0.07	0.01	0.07	0.02	0.13	0.02

a Total number of admissions at the center during the period of the study.

b Estimation of resident population (individuals) of the region during the wintering and breeding seasons according to the Catalan wintering bird Atlas 2009 and the Catalan breeding bird Atlas 1999-2002. Post-nuptial migration population is highly fluctuant and is not considered.

c Seasonal cumulative incidence (SCI) cases per 1000 animal/year =  [(total season cases^a^/estimated season population^b^)* 1000]/12.

d Number of individuals. Estimated population at the breeding season was calculated from the number of pairs multiplied by the number of chicks.

*Only species with at least up to 100 cases are represented in the table.

W =  Wintering period; B =  Breeding period.

### Inter-years distribution of specific causes of morbidity

**Figure 2:**
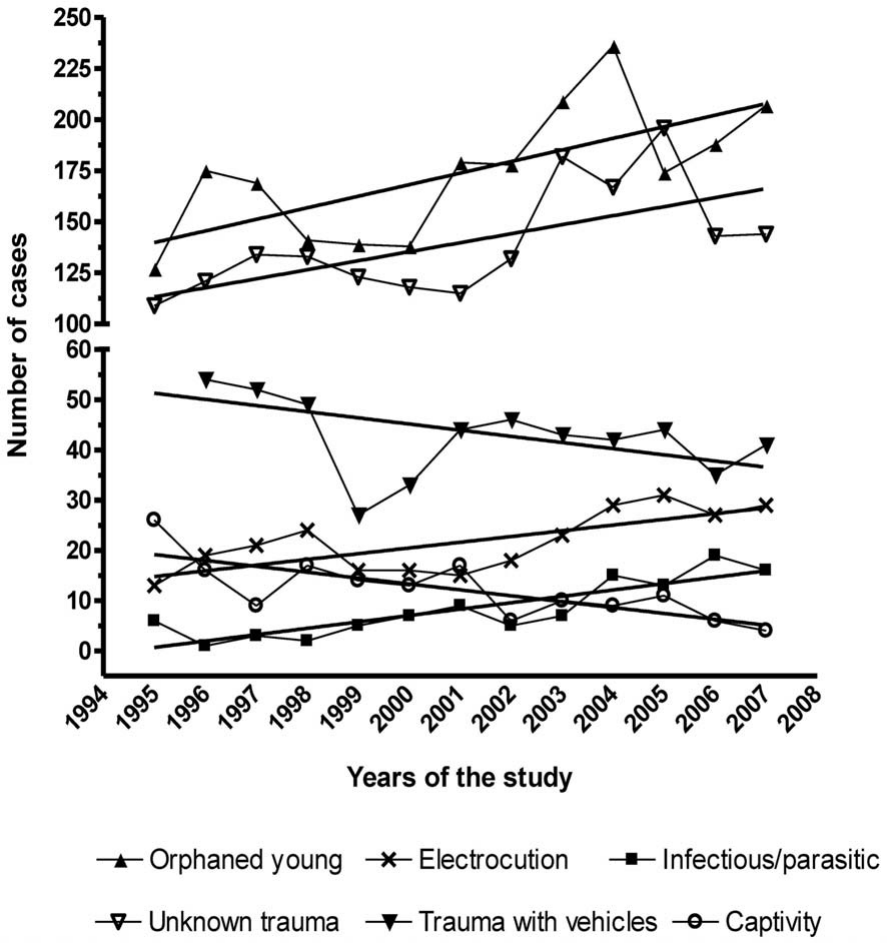
Different causes of admissions during the period 1995–2007 (number of cases). Only causes with significant statistical tendency are represented.

The number of admissions increased throughout the study period and a significant increase of cases was observed among the twelve years of the study in orphaned young birds, infectious/parasitic diseases, electrocution and unknown trauma. By contrast, a decreasing tendency was observed in the number of admissions due to undetermined cause, trauma with vehicles and captivity (Fig. 2).

## Discussion

Descriptive epidemiological studies of wildlife are an important source of information about natural and non-natural hazards to the wild animal population. In addition, studies of the causes of mortality and morbidity in wildlife have become an important source for ecosystem health monitoring [14], [15]. However, there are still important limitations of the information available due to lack of randomization, overrepresentation of human induced casualties, the heterogeneity of analytical methods [7], [16] and the low number of cases of free-living birds of prey reported [4], [5], [8], [17], [18]. Moreover, in most studies, disease frequency is estimated as a proportion of the cases of disease in the total number of admissions at the centres, lacking any information concerning the wild bird population and the particular risk for each species in the area of study.

The data presented in the current study were based on a large number of cases of very diverse wild raptor species, admitted to a wildlife rehabilitation centre during a long term period (12 years). Besides descriptive frequencies of morbidity cases admitted at the centre, the data included Seasonal Cumulative Incidences (SCI) based on the estimated wild raptor populations, for both wintering and breeding seasons. Thus, depending on the type of analyses performed, different information and conclusions can be obtained. Whereas, disease frequencies of morbidity entities (calculated as the proportion of each cause referred to the overall number of admitted cases) could allow a qualitative assessment of the hazards, the SCI (based on estimated wild raptor population) provides a more accurate approach to the potential ecological impact of the morbidity/mortality causes in the wild populations than the raw data.

Based on the present data it is evident that the anthropogenic origin was confirmed as the most frequent cause of hospitalization, comprising direct persecution (gunshot, poisoning, illegal captivity or traps) to involuntary human induced threats (collisions with vehicles, fences or electric lines and electrocution). Another clear finding was the high numbers of young orphaned cases admitted to the centre, which represented 32% of the total cases, and the fact that these cases increased throughout the study period. These values slightly differ from the ones reported by others [5], [17]. One of the most significant characteristics of this region is the large diversity of bird populations, in part due to its location within the migratory routes, in part to the great variety of habitats. On the other hand, this region is highly populated, and species with nesting areas close to urban settlements and other buildings are the most likely to be found and brought to the wildlife rehabilitation centres. In fact, *Falco tinnunculus* and *Otus scops,* the species with higher SCI for the orphaned category, use man-made structures to nest and so are directly exposed to anthropogenic interaction.

The analysis of data collected during the period of this study revealed that the number of animals with known information about age and gender increased in the later years of the study, indicating an improvement in data examination and collection by the centre. The high number of specimens with undetermined gender (67%), especially in birds belonging to the Strigiformes order, was mainly due to the high number of young or immature animals seen at the centre.

Trauma represented the main cause of admission (50% of cases, 60% when excluding orphaned bird category), with a prevalence similar to that described in other studies [4], [5], [8], [17]–[19]. The main source of traumas was either anthropogenic origin or unknown. The unknown trauma have been reported in very different proportions in the published reports, ranging from 32% of cases in *Accipiter nisus* [19] to 84% of cases in *Falco peregrinus* [20] and could be due to the different classification of the cases in the different studies.

Within the trauma category, gunshot represented the most common cause of admission (10% of the total). Although considerably lower than the 36% reported by Martínez et al. (2001) [8] in the East of Spain, it is of relevance that almost 10% of the casualties have been recorded out of the hunting season, as indication of deliberate prosecution. Even though birds of prey are legally protected species under Spanish law, shooting is still a major concern, especially in endangered species such as *Achila fasciata*, *Pandion haliaetus and Circus pygargus*. Interestingly, *Accipiter gentillis* and *Falco peregrinus* showed the highest SCI for gunshot. Both species have traditionally been considered as competing with small game hunters, and those values are again indication of deliberate shooting [21].

On the other hand, collision trauma with vehicle (8%) was the second highest cause of trauma, although in a lower proportion than previously reported in other studies [5], [17], [18]. This difference might be due to the high diversity of species analysed in the present study. Basically, the highest incidence of collision has been observed in owls -basically in *Athene noctua*, *Tyto alba* and *Strix aluco-* during the breeding and post-breeding period, which agrees with the results by Frías (1999) [22]. When we analyzed the SCI during the winter season, the highest risk was for *Tyto alba*, reinforcing the major vulnerability of this species for collision trauma [23]. In the Falconiformes order, *Buteo buteo* was the most affected species. This high risk could be related to its scavenging behaviour in the vicinity of roads. Moreover, we have also observed a winter peak of admissions in *Buteo buteo* and *Tyto alba*, possibly related to higher densities of these migratory species at this time of the year.

Another important cause of trauma was electrocution representing approximately 6% of the cases which is higher than studies in other areas [5], [17], [18]. The species distribution obtained in our study coincides with data published previously in Catalonia [24], [25]. *Bubo bubo* was the most affected owl with the highest SCI value, highlighting the potential impact of electrocutions in their wild population [9]. For diurnal raptors, the highest SCI was for *Accipiter gentillis* during winter and *Buteo buteo* in the breeding season. Both species have similar anatomical features that make them highly vulnerable to electrocution. On the other hand, we found a higher percentage of electrocutions in *Falco tinnunculus* compared to a previous report in Spain [26]. Despite the small size of this falcon, the perching behaviour of this species is a well-known risk factor that could explain the present results.

Captivity of birds of prey, especially Falconiformes, is still an important cause of admission in Spain [8]. However, the frequency was clearly lower than the 18% reported by Martínez et al. (2001) [8]. Noteworthy, the most commonly captive species was Falco *tinnunculus -*mostly related with illegal trade of birds and falconry- followed by *Athene noctua* and *Otus scops*. Both owl species were probably captured when young birds and kept as pets in captivity. Finally, the proportion of undetermined causes showed similar values to other retrospective surveys [11], [18], [20], indicating that the lack of obtaining a specific diagnosis in birds of prey is around 10% of the total admissions.

Data from rehabilitation centres based on live birds is useful for detecting primary infectious or parasitic diseases. Digestive tract disease caused by *Trichomonas gallinae* was the most frequent disease observed in both diurnal raptors and owls, in agreement with Wendell et al. (2002) [5].

Trichomoniasis was diagnosed by both direct examination and cytology (Diff-Quick stained) of scraping of oral or upper digestive tract lesions. Since we have focused our study in primary causes of admissions, the role of underlying infectious or parasitic diseases has been underestimated, because of no complete microbiological and parasitological analyses were done routinely in all cases due to financial constraints, autolysis or the statement of a primary diagnosis. Finally, intoxication was anecdotally included in our study due to financial limitations in the diagnosis.

Analysis of the principal causes of morbidity throughout the twelve years of study showed a decrease in the undetermined cause category that could be an indication of an improvement in the quality of the diagnostic protocols and staff experience. Similarly, the increase of hospitalized cases by electrocution and the decrease of casualties by captivity could be explained by increased efficiency of the wildlife police services. As suggested above, the increase of cases in the young orphaned category could be related to both the human demographic traits of Catalonia and a better knowledge by the inhabitants about the role of wildlife rehabilitation centres. On the other hand, the increased cases by unknown trauma could be due to a greater participation of people taking care of injured animals, but also suggest the difficulty in the trauma classification. Another interesting finding was that gunshot fatalities have become stable over the years, pointing out the enormous deficiencies in the police investigative process and the necessity of stronger legal action from the relevant authorities.

In conclusion, the long term epidemiological research conducted at the wildlife rehabilitation center determined the main environmental and anthropogenic causes of morbidity in wild raptor populations of Catalonia. In addition, the weight of different epidemiological markers such as the seasonal cumulative incidence can provide more accurate statistics about the dynamics of wild raptor populations in the studied area.

## Materials and Methods

### Study design and animals

A retrospective study was performed using the original medical records of birds of prey admitted at the Wildlife Rehabilitation Centre of Torreferrussa from 1995 to 2007. The centre receives animals from all of Catalonia (North-East Spain, 3°19′-0°9′ E and 42°51′-40°31 N), mainly from the South and Central areas. More than thirty species of diurnal raptors and eight different owl species have been observed in this area, most of which are breeding species [27].

The centre directly depends on the governmental Catalan Wildlife-Service. Thus, protocols, amendments and other resources were done according to the guidelines approved by the government of Catalonia.

### Definition of variables

Species, gender, age, date and primary cause of admission were included in the data analyses. Sex was determined when possible by inspection in dimorphic species [28] or by gonadal examination at necropsy. Age was categorized as “first year calendar” and “>1 year calendar” according to Martínez et al. (2001) [8]. The year was divided into three seasons: breeding (from March to July), post-nuptial migration (August to October) and wintering (November to February).

Our general classification of primary morbidity causes was adapted from different studies [4], [5], [29] as follows: trauma, infectious/parasitic disease, metabolic/nutritional disease, toxicosis, orphaned young birds, and unknown/undetermined. Two more categories of causes were added: captivity and fortuity. The captivity category included wild birds maintained illegally in captivity for more than 6 months and the fortuity category included all animals with no associated medical primary cause (birds found inside buildings, farms, water ponds, entangled in plants or manure heaps). The orphaned young category integrated chicks and fledging raptors (Table 2). To assign these categories we used different information obtained from different sources: (a) the physical examination performed by the veterinarian at the admission instance; (b) the anamnesis of people that recovered the bird; (c) the medical reports or case history; and when possible (d) from complementary diagnostic tools, as now radiography (basically to corroborate gunshots), blood chemistry and haematology, cytology and toxicology. Post-mortem diagnoses were done when birds arrived dead to the centre, when they had to be euthanized for bad prognoses or died due to the primary cause.

The trauma category was subdivided into: collision, electrocution, gunshot, trap, predation, and unknown trauma (for those cases with clinical signs of trauma but without clear information about the circumstances of the accident). Collision traumas were further subdivided into impacts with motor vehicles, buildings, power lines, fences, and others. The diagnosis of electrocution was based on the information recorded in the anamnesis and the clinical signs (presence of electric burns mainly affecting feathers, skin and soft tissues).

The metabolic and nutritional disorder category comprised birds with low body condition or weakness, suffering from metabolic bone diseases (MBD) and the rest of diagnoses were grouped by organic systems (Table 2). The infectious disease category was applied when a pathogenic microorganism was confirmed by microbiological, parasitological or histopathological diagnosis.

### Statistical analysis

Descriptive statistics, normality test and inferential analyses were done at 95% of confidence with SPSS Advanced Models ™ 15.0 (SPSS Inc. 233 South Wacker Drive, 11th Floor Chicago, IL 60606-6412). Chi-square (χ^2^) or Fisher exact tests were used for comparison between proportions. Odds ratio measure of association was employed for disease comparisons. Seasonal cumulative incidences (SCI) were calculated for the wintering and breeding seasons, and were defined as the number of cases per season divided by the estimated population at that season. Results were expressed per 1000 animal and year. Reference populations of the region were obtained from published data [27], [30]. Breeding and wintering estimated populations were considered as stable during the seasons and over the period of study. Trend analyses were applied for specific causes with a minimum of 100 cases in order to detect differences among years.

## References

[1] Kovács A, Mammen UCC, Wernham CV (2008). European Monitoring for Raptors and Owls: State of the Art and Future Needs.. Ambio.

[2] Sergio F, Newton I, Marchesi L, Pedrini P (2006). Ecologically justified charisma: preservation of top predators delivers biodiversity conservation.. J Appl Ecol.

[3] Burfield IJ (2008). The Conservation Status and Trends of Raptors and Owls in Europe.. Ambio.

[4] Morishita TY, Fullerton AT, Lownestine L, Gardner IA, Brooks DL (1998). Morbidity and mortality of free-living raptorial birds of Northern California: a retrospective study, 1983-1994.. J Avian Med Surg.

[5] Wendell MD, Sleeman JM, Kratz G (2002). Retrospective study of morbidity and mortality of raptors admitted to Colorado state university veterinary teaching hospital during 1995 to 1998.. J Wildl Dis.

[6] Hernández M (1988). Road mortality of the little owl (*Athene noctua*) in Spain.. J Raptor Res.

[7] Real J, Grande JM, Mañosa S, Sánchez-Zapata JA (2001). Causes of death in different areas for Bonelli's eagle *Hieraetus fasciatus* in Spain.. Bird Study.

[8] Martínez JA, Izquierdo A, Zuberogoitia I (2001). Causes of admisión of raptors in rescue centres of the East of Spain and proximate causes of mortality.. Biota.

[9] Martínez JA, Martínez JE, Mañosa S, Zuberogoitia I, Calvo F (2006). How to manage human-induced mortality in the Eagle Owl *Bubo bubo*.. Bird Conserv Int.

[10] González LM, Margalida A, Mañosa S, Sánchez R, Oria J (2007). Causes and spatio-temporal variations of non-natural mortality in the Vulnerable Spanish imperial Eagle *Aquila adalberti* during a recovery period.. Oryx.

[11] Newton I, Wyllie I, Dale L (1999). Trend in the number and mortality patterns of sparrohawks (*Accipiter nisus*) and kestrels (*Falco tinnunculus*) in Britain, as revealed by carcass analyses.. J Zool.

[12] Rodríguez B, Rodríguez A, Siverio F, Siverio M (2010). Causes of raptor admissions to a wildlife rehabilitation Center in Tenerife (Canary Islands).. J Raptor Res.

[13] Dirección General para la Biodiversidad. Ministerio de Medio Ambiente (2006). Catálogo Nacional de Especies Amenazadas (1990-2006)..

[14] Brown JD, Sleeman JD (2002). Morbidity and mortality of reptiles admitted to the Wildlife Center of Virginia, 1991 to 2000.. J Wildl Dis.

[15] Sleeman JM, Fowler ME, Miller RE (2008). Use of Wildlife Rehabilitation Centers as monitors of ecosystem health.. Zoo and Wild Animal Medicine.

[16] Newton I, Cooper JE (2002). Diseases in wild (free-living) raptors.. Birds of prey.

[17] Deem SL, Terrell SP, Forrester DJ (1998). A retrospective study of morbidity and mortality of raptors in Florida: 1988-1994.. J Zoo Wildl Med.

[18] Komnenou AT, Georgopoulou I, Savvas I, Dessiris A (2005). A retrospective study of presentation, treatment, and outcome of free-ranging raptors in Greece (1997-2000).. J Zoo Wildl Med.

[19] Kelly A (2006). Admissions, diagnoses, and outcomes for Eurasian sparrowhawks (Accipiter nisus) brought to a wildlife rehabilitation center in England.. J Raptor Res.

[20] Harris MC, Sleeman JM (2007). Morbidity and mortality of bald eagles (Haliaeetus leucocephalus) and peregrine falcons (Falco peregrinus) admitted to the Wildlife Center of Virginia, 1993-2003.. J Zoo Wildl Med.

[21] Mañosa S (2002). The conflict between game bird hunting and raptors in Europe.. Unpublished report to REGHAB Project.

[22] Frías O (1999). Seasonal dynamics of avian traffic casualties on Central Spain: age and number of individuals and species richness and diversity.. Ardeola.

[23] Martínez JA, Zuberogoitia I (2004). Habitat preferences and causes of population decline for barn owls *Tyto alba*: a multi-scale approach.. Ardeola.

[24] Mañosa S (2001). Strategies to identify dangerous electricity pylons for birds.. Biodiversity Conserv.

[25] Tintó A, Real J, Mañosa S (2010). Predicting and correcting the electrocution of birds in mediterranean areas.. J Wildl Manage.

[26] Guzmán J, Castaño JP (1998). Raptor mortality by electrocution in power lines in eastern Sierra Morena and Campo de Montiel (Spain).. Ardeola.

[27] Estrada J, Pedrocchi V, Brotons L, Herrando S (2004). Catalan breeding bird Atlas 1999-2002. Institut Català d' Ornitologia (ICO).. Lynx Editions.

[28] Baker K (1993). Identification Guide to European Non-Passerines..

[29] Samour JH (2004). Causes of morbidity and mortality in Falcons in Saudi Arabia.. J Avian Med Surg.

[30] Herrando S, Brotons L, Estrada J, Guallar S, Anton M (2011). Atles dels ocells de Catalunya a l'hivern 2006-2009.. Lynx Editions In Press.

